# Perception of Noise Pollution Among Youths and Adults in Urban Puducherry, South India

**DOI:** 10.7759/cureus.49573

**Published:** 2023-11-28

**Authors:** Debajyoti Bhattacharya, James TD, Subitha Lakshminarayanan, Sai Meenu, Swathy Madhusoodanan L, Mahalakshmy Thulasingam

**Affiliations:** 1 Preventive and Social Medicine, Jawaharlal Institute of Postgraduate Medical Education and Research (JIPMER), Puducherry, IND

**Keywords:** traffic noise, hearing loss, annoyance, noise sensitivity, noise pollution

## Abstract

Objective

The study seeks to assess the perceptions of people on evaluating the sources of noise, noise-induced health issues, and noise regulation awareness among the exposed population present in the study site of urban Puducherry, South India.

Methods

A cross-sectional survey using a pre-tested semi-structured questionnaire was conducted between July and August 2021 in 32 study sites in urban Puducherry to evaluate how adults and youth perceive noise pollution. The questionnaire gathered details on their sociodemographic characteristics, knowledge of the problems associated with noise pollution, source of noise pollution, effects of noise on health, and awareness of regulations related to noise pollution.

Results

Half of the study participants perceive that noise pollution is a problem in their localities; the majority feel disturbed by that noise; and the most prevalent reason given for noise pollution is traffic noise. Most of the participants reported that trouble paying attention to work or conversations was the most frequent health impact of noise pollution. Participants who are employed, have formal education, belong to families above the poverty line, and reside near the main road and sub-main road (less than 200 meters) showed a significant association (p-value <0.05) with perceived noise pollution problems.

Conclusion

Based on the findings, it can be concluded that respondents in urban Puducherry perceive traffic noise as the most common source of noise pollution. The majority of the participants felt that the excessive noise made it difficult for them to focus on their work. Individuals who reside or work close to a major highway and outdoor workers believe noise pollution is a big problem.

## Introduction

Health literacy is a growing concern in developing as well as developed countries and is essential to making effective decisions and interventions across various health and environmental domains. Environmental health literacy has been an emerging field since the 1950s. Kollmuss acknowledged the gap in knowledge, awareness, and pro-environmental behavior and attributed demographic, social, economic, and cultural factors associated with environmental health [1]. The World Health Organization (WHO) recognized noise as a contaminant in 1972 [2] and defined it as unwanted sound generated by human activity above 65 decibels (dB) [3]. Noise is one of the primary contributors to pollution in urban areas, is the third-most harmful pollutant after air and water, and can have both auditory and non-auditory health impacts if it exceeds 75 dB [3]. Globally, 1 billion young individuals are at risk of developing permanent, preventable hearing loss [4], and more than 5% of people worldwide need rehabilitation for their "disabling" hearing loss (432 million adults and 34 million children). Over 700 million people, or one in 10 people, are predicted to have hearing loss that is temporarily incapacitated by the year 2050 [4].

The WHO has identified seven categories of human health consequences caused by noise pollution: (1) hearing impairment, (2) interference with spoken communication, (3) sleep disturbances, (4) cardiovascular disturbances, (5) disturbances in mental health, (6) impaired task performance, and (7) negative social behavior and annoyance reactions [5]. The burden of disease analysis done in developed countries shows that one out of every 100 premature deaths is caused by transportation noise. The data shows the alarming situation and provides insights into how severe noise pollution is in developed countries [6].

Studies show workers exposed to excessive noise levels are at increased risk for ischemic heart disease (IHD), stroke [7], hypertension [8], annoyance, poor sleep quality, and sensitivity to noise [9] among residents living near highways [10]. Those with a higher exposure to transportation noise are associated with a higher risk for dementia [11] and Alzheimer's [12,13]. Traffic noise also contributed to reading comprehension, recall, and annoyance [14], and specifically tinnitus and hearing loss among traffic personnel [15]. It is a source of irritation among residents at several silent, residential, commercial, industrial zones, and road intersections [16] and causes headaches, poor tempers, hearing problems, loss of concentration, and sleep disruption due to background noise [17,18].

Various studies support the importance of both subjective and objective noise pollution [19-22]. Utilizing observational tools and assessing people's perception levels, noise pollution could be measured. Observed noise pollution can be linked to perceived noise pollution [19]. People's mobility preferences, perceived overall health, feeling of well-being, and overall quality of life are all impacted by how they perceive noise pollution [19,20]. However, perceived noise pollution has a significant impact on people's health [20,22,23]. To improve people's perceptions of their health, general well-being, and contentment, it is essential to investigate perceived noise pollution [19]. Residents' discomfort is rarely acknowledged, even if their perceptions of noise are identical to the levels that have been recorded [21,22,24].

Self-reported health is related to a person's mental, social, and physical well-being, which cannot be measured by a single morbidity measure [23]. Low self-reported health is significantly associated with premature death because it is intrinsically tied to this wider picture of health. The range of health conditions driven by numerous social, economic, and environmental factors, however, may be found in self-reported health surveys. In most cases, self-reported health questionnaires, for example, start with a single, straightforward question and end with three to five evaluations (poor, medium, or good health conditions). Environmental issues like noise pollution and one's socioeconomic position may have an impact on one's health [19].

There is a paucity of literature on the perception of noise pollution among youths and adults in the South Indian region. The study seeks to assess the perceptions of people about evaluating the sources of noise, noise-induced health issues, and noise regulation awareness among the exposed population present in the study site of urban Puducherry, South India.

## Materials and methods

Study design and participants

A cross-sectional analytical study was conducted from July to August 2021 in Puducherry, an Indian Union Territory that lies on the southeastern coast of India. The total population of Puducherry district as per the Census of India 2011 was 9,50,289, of which males and females were 468,258 and 482,031, respectively [25]. Urban Puducherry city includes Oulgaret municipality (24.1 km²) and Puducherry municipality (17.8 km²). The average literacy rate in urban Puducherry is 88.5%, with a population density of 3232 per sq. km [25]. Youth and adults residing in or passers-by in urban Puducherry, i.e., Puducherry and Oulgaret municipality, were enrolled in the study. Ethical approval for this study was obtained from the Jawaharlal Institute of Postgraduate Medical Education and Research (JIPMER) Institute Ethics Committee (approval no. JIP/IEC/2021/0147) and the Post Graduate Research Monitoring Committee (PGRMC).

Sample size and sampling

**Figure 1:**
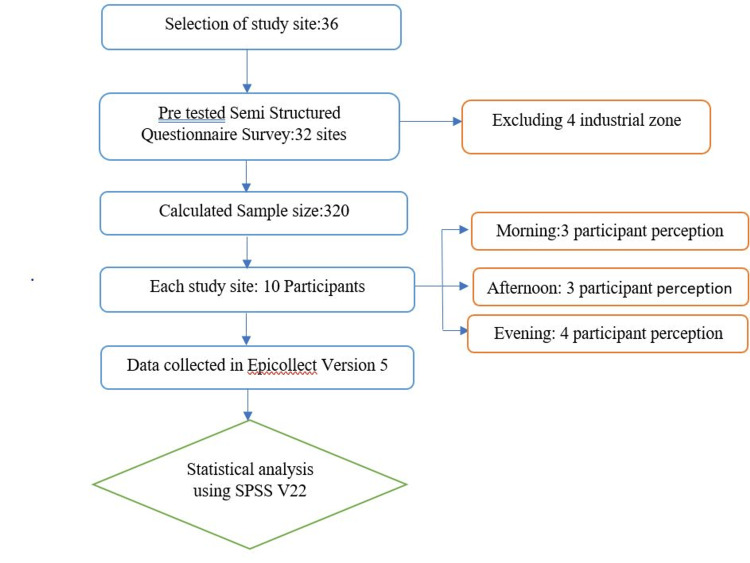
Methodology employed to enroll and evaluate study participants

The sample size was determined using OpenEpi software version 3.01 (a free and open-source software for epidemiologic statistics) with an expected prevalence of 40% [26], a margin of error of 6%, and a 95% confidence interval. The initial estimate was 257, and after accounting for a non-response rate of 20%, the final sample size was rounded to 320. Figure 1 illustrates the sampling process involved in selecting 10 participants from each of the 32 study locations (10 study sites each from commercial and silent zones, and six study sites each from residential zones and traffic junctions), excluding the industrial zone out of a total of 36 locations [27]. The inclusion criteria encompassed individuals aged 18 or older who reside, work, or were passing by the study site. The exclusion criteria comprised individuals who declined to participate and non-residents of the Union Territory.

Study tools

The semi-structured questionnaire's design was accomplished by utilizing previous studies and consulting experts. To validate the questionnaire, a pilot study among college students was conducted. Five components make up the final questionnaire (available in local language and English), including sociodemographic characteristics, knowledge of noise problems, knowledge of the sources of noise, awareness of noise-related regulations, and knowledge about the health effects of noise pollution [13,28-31]. The questionnaire was used to evaluate the perception of youths and adults of urban Puducherry in 32 study sites after written consent and brief details about the purpose of the study, and its benefits, and then verbally interviewing them in the local language. The questionnaire gathered details such as socio-demographic characteristics like age, gender, religion, poverty status, educational status, occupational status, and working environment. The questionnaire focused on understanding noise pollution, its sources, and its effects on individuals. It also asked about the perceived source of noise pollution, such as traffic or religious places, and the time spent experiencing it. It also explored knowledge about noise pollution rules and regulations, as well as the health effects of noise pollution, such as hearing loss and hearing problems.

Data analysis

Data collection was performed through EpiCollect version 5 (Imperial College, London, UK) and exported in comma-separated value (CSV) format. Subsequently, data analysis was conducted using SPSS version 22 (IBM Corp., Armonk, NY, USA). Within the analytical framework, categorical variables were employed as independent variables, while the occurrence of noise pollution issues within the study area was examined as the dependent variable.

## Results

Table 1 provides an overview of the study's demographic composition. Among 320 participants, 172 (53.7%) are male, with a median (IQR) age spanning from 31 to 52 years. The majority of participants 276 (86.3%) identify as Hindus, and over 177 (55.3%) of them fall below the poverty threshold. Notably, only 34 (11%) of the individuals have no formal education, and 273 (85.3%) of the employed participants work in an outdoor setting. Additionally, more than half the respondents 177 (55.3%) have their residences or workplaces situated within 200 meters of a highway, main road, or sub-major road, while 202 (63.1%) live or work in proximity to the main road.

**Table 1:** Sociodemographic and environmental characteristics of youths and adults in urban Puducherry, South India, 2021 Note: The data has been represented as n, % #Poverty status is based on the color of the ration card *Working environment is only applied to those who are employed

Characteristics	Frequency (n)	Percentage (%)
Total	320	
Age in years (median IQR)	40.0 (31.0-52.0)	
Gender		
Male	172	53.7
Female	148	46.3
Religion		
Hindu	276	86.3
Muslim	30	9.4
Christian	13	4.0
Jainism	1	0.3
Poverty Status #		
Below poverty line	177	55.3
Above poverty line	143	44.7
Educational Status		
No formal education	34	10.6
8^th^ standard	68	21.2
10^th^ standard	45	14.1
12^th^ standard	45	14.1
Graduate	95	29.7
Postgraduate	33	10.3
Occupational Status		
Employed	273	85.3
Unemployed	29	9.0
Homemaker	7	2.2
Students	7	2.2
Retired	4	1.3
Working Environment n=277*		
Outdoor	186	67.1
Indoor	91	32.9
Road type near to your place		
Main road	202	63.1
Highway	71	22.2
Sub-main road	47	14.7
Distance of road near to your place		
≤200 meters	177	55.3
>200 meter	143	44.7

Table 2 illustrates that of the 320 participants, more than two-thirds, i.e., 216 (67.5%), are aware of noise pollution. Of these 216 participants, more than half, i.e., 167 (52.2%), feel noise pollution is an issue in their place. The majority of these 167 participants, i.e., 154 (92.2%), feel that noise pollution is a problem in their neighborhood. Among this majority of 154 participants, the most common sources are considered to be traffic and outdoor noise (154, 100%), followed by other noise (76, 49.4%), religious place noise (17, 11%), and workplace noise (15, 9.7%). Of these 154 participants, 109 (70.8%) find the mornings noisier, more than half of the respondents (96, 62%) are not concerned about noise pollution legislation, 146 (94.8%) believe that traffic flow has increased in recent years, 24 (15.6%) admit to discussing noise pollution with neighbors, and more than half of the respondents (89, 57.8%) plan on filing complaints of noise pollution in the future.

**Table 2:** Perception about the source of noise pollution and awareness of noise-related rules and regulations among youth and adult participants affected by noise pollution in urban Puducherry Note: The data has been represented as n, %

Characteristics	Frequency (n)	Percentage (%)
Awareness about noise pollution n=320	216	67.5
Noise pollution problem in your place n=320	167	52.2
Feeling disturbed due to noise pollution n=167	154	92.2
Source of noise pollution #Outdoor	154	100.0
Traffic Noise	154	100.0
Others (fireworks/meetings/loud music)	76	49.4
Religious place noise near your place	17	11.0
Workplace noise	15	9.7
Time in which you experience noise pollution		
Morning	109	70.8
Evening	41	26.7
Other times	4	2.5
Awareness of noise pollution rules and regulations	58	37.7
Increase in the flow of traffic in recent times	146	94.8
Prior engagement in conversation with your neighbor about noise pollution	24	15.6
Ever lodged a complaint about a neighbor being too noisy?	10	6.5
In the future, would you lodge a complaint about a neighbor being too noisy?	89	57.8

Table 3 illustrates that of the 154 participants who feel disturbed by noise pollution, 10 (7%) who experienced prior noise disturbance acknowledge hearing loss-related issues. The most common health effects associated with noise pollution perceived by these 154 participants are difficulty paying attention to work or conversations (130, 84.4%), followed by noise sensitivity (112, 72.7%), headaches (63, 40.9%), feelings of anxiety (57, 37%), and tinnitus or ringing in the ears (53, 34.4%).

**Table 3:** Perception of health effects of noise pollution among the 154 youth and adult participants who feel disturbed due to noise pollution in urban Puducherry Note: The data has been represented as n, %

Perceived health effects of noise pollution	Frequency (n)	Percentage (%)
Ear pain	23	14.9
Difficulties paying attention to work/conversation	130	84.4
Noise sensitivity	112	72.7
Headache	63	40.9
Feeling anxious	57	37.0
Tinnitus or ringing in the ear	53	34.4
Sleep disturbance	44	28.6
Feel depressed	22	14.3
Hearing loss/hearing problems	10	6.5
Dizziness	10	6.5
Hearing aids use	4	2.6

Table 4 illustrates that out of the total 320 participants, 167 (56.9%) have a noise pollution problem near their residence or workplace. Noise pollution is perceived as a problem among the employed (unadjusted prevalence ratio (PR) 1.6, 95% CI 1.0-2.5; p = 0.006). Participants with formal education (unadjusted PR 1.5, 95% CI 0.9-2.4; p = 0.037) and above the poverty line (unadjusted PR 0.6, 95% CI 0.4-0.7; p = <0.001) have a better perception of noise pollution. Study participants who reside near the main road and sub-main road (unadjusted PR 0.6, 95% CI 0.5-0.7; p = <0.001) and close to a distance of fewer than 200 meters (unadjusted PR 5.6, 95% CI 3.7-8.3; p = <0.001) from the highway have higher noise exposure compared to those who reside away from the highway and those who reside at further distances from the highway, which is statistically significant.

**Table 4:** Association of sociodemographic characteristics with perceived noise pollution problems among youth and adult participants in urban Puducherry Note: The data has been represented as n, %, and prevalence ratio (95%) CI Confidence interval calculated using OpenEpi *p-value is considered statistically significant (p <0.05, p <0.00)

Characteristics	Total	Noise pollution percieved as a problem		Prevalence ratio (95%) CI	P-value*
Total	320	Yes (167)	No (153)		
Age (years)		n (%) n (%)			
≤40	167	95 (56.9)	72 (43.1)	1.2 (0.9-1.4)	0.079
>40	153	72 (47.1)	81 (52.9)		
Gender					
Male	172	96 (55.8)	76 (44.2)	1.1 (0.9-1.4)	0.162
Female	148	71 (48.0)	77 (52.0)		
Religion					
Hindu	276	149 (54.0)	127 (46.0)	1.3 (0.9-1.9)	0.107
Others	44	18 (40.9)	26 (59.1)		
Poverty Status					
Below poverty line	177	72 (40.7)	105 (59.3)	0.6 (0.4-0.7)	<0.001*
Above poverty line	143	95 (66.4)	48 (33.6)		
Educational Status					
Formal	286	155 (54.2)	131 (45.8)	1.5 (0.9-2.4)	0.037*
No formal education	34	12 (35.3)	22 (64.7)		
Occupational Status					
Employed	277	153 (55.2)	124 (44.8)	1.6 (1.0-2.5)	0.006*
Unemployed	43	14 (32.6)	29 (67.4)		
Road type near to your place					
Main road & Sub-main road	249	115 (46.2)	134 (53.8)	0.6 (0.5-0.7)	<0.001*
Highway	71	52 (73.2)	19 (26.8)		
Distance of road near to your place					
≤ 200 meters	177	146 (82.5)	31 (17.5)	5.6 (3.7-8.3)	<0.001*
>200 meters	143	21 (14.7)	122 (85.3)		

## Discussion

The findings of this study reveal that two-thirds of the respondents are aware of noise pollution, and half of them feel noise pollution in their area. Outdoor noise contributes to the majority of noise pollution, and traffic noise is the key contributor to that. The majority of the respondents feel disturbed by that noise. In comparison to other times of the day, the morning is noisier because of more traffic during the rush hour, including school and college buses, office buses, three-wheeler autos, and regular public transport vehicles. More than half of the participants are not aware of noise pollution rules and regulations. Among non-auditory health effects, difficulty paying attention to work or conversation was perceived as the highest by the respondents, followed by headaches, feelings of anxiety, sleep disturbances, depression, and dizziness. Noise sensitivity is perceived as the highest among the auditory health effects, followed by tinnitus, prior reading or hearing about hearing loss problems, ear pain, hearing loss, and hearing aid use. Employed individuals perceive noise pollution problems more than the unemployed because most of them work in outdoor environments. Respondents with formal education have a higher perception of noise pollution problems compared to those without any formal education. The prevalence of noise pollution problems is higher among those who reside within 200 meters proximate to the road than among those who reside more than 200 meters proximate to the road (82.5% vs. 14.7%).

A similar study was done on 3000 individuals in Finland within the age group of 25 to 74 years, and the perception of 80% of respondents shows they were exposed to road traffic noise in their residences at some point, and 18% reported they were exposed to high or excessive levels. Similarly, 13% of respondents stated they were exposed to traffic exhaust in their homes at a high to an excessive level, and 41.7% of those respondents said they were annoyed to some extent [11]. A Bangladesh study shows that 91% of respondents stated their neighborhood was noisy, and road traffic and construction activities were identified as the main sources of noise pollution [32]. The occupational status of respondents shows statistical significance with noise sensitivity [33].

Research has shown that noise annoyance is more related to road traffic as compared to rail or air traffic [34]. In a study conducted in Srinagar Municipal Corporation, 565 participants in 41 measurement locations were interviewed with a semi-structured questionnaire that revealed traffic noise was reported by 85% of respondents at home, and approximately 64% of those surveyed considered traffic noise to be extremely loud [9]. According to the study by Joseph et al., 62.1% of audiologists and 49.1% of the general population believe that hospital staff members are in danger of being impacted by noise at work, which may have negative effects on their ability to focus, regulate their emotions, or perform their jobs efficiently [35]. The research findings of Yang underscore a noticeable urban-rural disparity in the perception of noise pollution [36]. In a study by Agarwal and Swami, approximately 52% of the study population reported experiencing frequent irritability, with 48.6% attributing sleep disturbances to noise pollution [10]. Notably, a significant 84% of the respondents expressed annoyance caused by traffic noise, and 62.3% reported disruptions in their sleep due to traffic noise. Furthermore, the study identified statistically significant correlations between residing in noisy environments and the experience of irritation stemming from traffic noise (p < 0.001) [9]. These findings emphasize the substantial impact of noise pollution on the well-being and comfort of individuals, particularly in urban settings.

For an effective response to noise pollution, a collaborative approach is essential on the part of the government. This involves facilitating the exchange of accurate information, fostering a supportive attitude toward noise reduction initiatives, and proactively implementing measures. Governments should engage in coordinated campaigns, social mobilization efforts, and communication strategies alongside routine noise pollution surveillance. These activities serve to educate and equip communities to address this critical public health concern. In addition, the development and execution of policies should take into account varying levels of literacy within the population and utilize platforms such as social media and television to disseminate public information about local noise pollution management strategies.

The WHO has launched the Integrated People-Centred Ear and Hearing Care (IPC-HC) initiative, aimed at providing universal access to high-quality ear and hearing care services across the lifespan. They encapsulate the core components of this initiative in an acronym: *h*earing screening and intervention, *e*ar disease prevention and management, *a*ccess to technologies, *r*ehabilitation services, *i*mproved communication, *n*oise reduction, and *g*reater community engagement (HEARING) [4]. This comprehensive approach addresses the multifaceted aspects of ear and hearing care to enhance the well-being of individuals and communities.

This baseline study investigates the association between noise pollution perceptions and self-reported health status among urban populations in South India. The research reveals that individuals with formal education tend to view noise pollution as a more significant concern, with traffic noise as the primary source. The most commonly reported health impact is difficulty concentrating at work or in conversations. Furthermore, employed individuals, especially those working outdoors and those residing or working near major roadways, exhibit a heightened awareness of noise pollution as a major issue compared to individuals who spend more time at home or live farther from major roads.

As per our knowledge, this is the first study in South India to cover respondents from different zones about noise pollution. We have several strengths in this study by using a semi-structured Tamil questionnaire to assess knowledge about noise pollution problems, sources, health effects, and awareness of rules. The study's limitation lies in its reliance on self-reported health assessments by youth and adults in urban Puducherry, which is not the same as a clinical diagnosis. Moreover, the study was conducted at the end of the second wave of the COVID-19 pandemic, and there were restrictions on the movement of people.

## Conclusions

In a broader context, this study emphasizes the crucial influence of education, socioeconomic status, and proximity to major roadways in shaping individuals' perceptions of noise pollution and its resulting health implications. Those with higher literacy levels are more likely to recognize noise pollution as a significant concern, particularly in urban areas where traffic noise is a major problem. Sociodemographic factors, encompassing employment, formal education, above-poverty-line status, and living near major roadways, are associated with a heightened awareness of noise pollution issues and correlated health problems.
